# Insights on the Modulation of SIRT5 Activity: A Challenging Balance

**DOI:** 10.3390/molecules27144449

**Published:** 2022-07-12

**Authors:** Matteo Mori, Giulia Cazzaniga, Fiorella Meneghetti, Stefania Villa, Arianna Gelain

**Affiliations:** Department of Pharmaceutical Sciences, University of Milan, Via L. Mangiagalli 25, 20133 Milano, Italy; matteo.mori@unimi.it (M.M.); giulia.cazzaniga@unimi.it (G.C.); fiorella.meneghetti@unimi.it (F.M.); arianna.gelain@unimi.it (A.G.)

**Keywords:** Sirtuin 5, HDACs, activators, inhibitors, natural compounds, small molecules

## Abstract

SIRT5 is a member of the Sirtuin family, a class of deacetylating enzymes consisting of seven isoforms, involved in the regulation of several processes, including gene expression, metabolism, stress response, and aging. Considering that the anomalous activity of SIRT5 is linked to many pathological conditions, we present herein an overview of the most interesting modulators, with the aim of contributing to further development in this field.

## 1. Introduction

Class III histone deacetylases (HDACs), commonly known as Sirtuins (SIRTs), are NAD^+^-dependent enzymes characterized by either histone deacetylase or, more frequently, ribosyl transferase activity [1]. SIRTs are involved in several processes, including transcription, translation, metabolism, and cellular stress/damage. They catalyze different types of chemical reactions, such as demalonylation, desuccinylation, depropionylation, and delipoamidation [2]. Besides their role in gene expression and metabolism, SIRTs are implicated in delaying cellular aging and controlling the normal cell cycle; moreover, they are important mediators of apoptosis inhibition and glucose homeostasis. Interestingly, the downregulation of these enzymes has been associated with metabolic diseases, cancer, and neurodegeneration.

SIRTs comprise seven isoforms, sharing a conserved catalytic core and differing in their N- and C-terminal sequences, which contribute to their specific localization and regulation. SIRT1 has an N-terminal STAC (sirtuin-activating compound) binding domain, SIRT3, 4, and 5 are characterized by a peculiar N-terminal mitochondrial localization sequence (MLS), while SIRT6 and SIRT7 have N- and C-terminal portions contributing to the binding of DNA and chromatin [3].

**Scheme 1:**
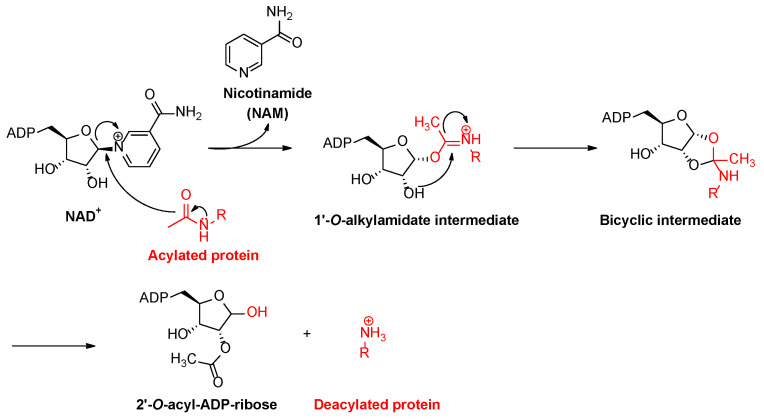
Mechanism of the deacetylation reaction catalyzed by SIRTs.

The different SIRT isoforms catalyze the same deacetylation reaction: NAD^+^ reacts with the acyclic oxygen of the protein substrate to form a 1′-*O*-alkylamidate intermediate; then, subsequent hydrolysis provides the deacylated protein and 2′-*O*-acyl-ADP-ribose. In this reaction, Zn^2+^ plays a fundamental role as an enzymatic cofactor (Scheme 1) [3].

**Figure 1:**
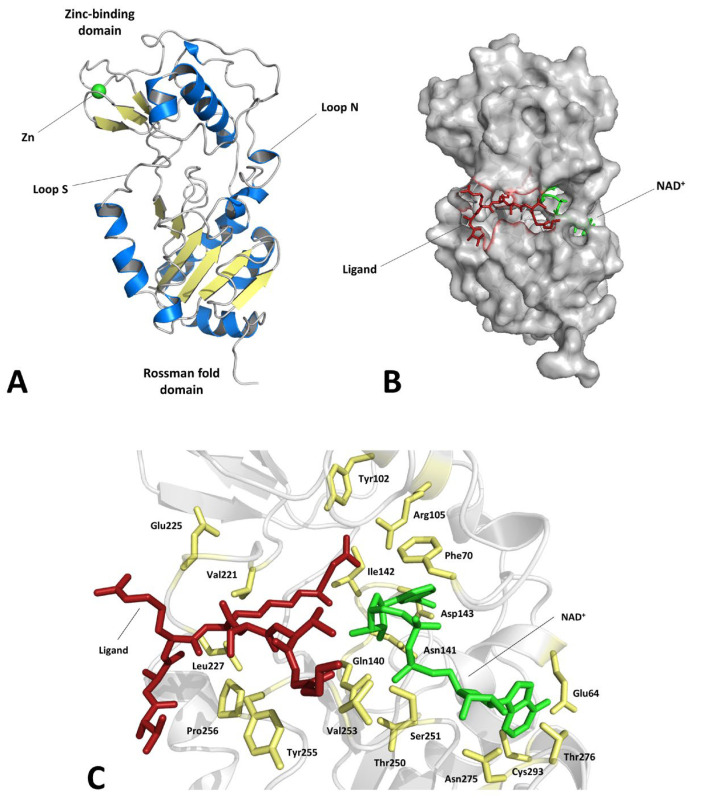
(**A**) Ribbon diagram of SIRT5 (PDB code: 3RIY), evidencing the two domains and the position of the zinc cofactor. α-Helices are colored blue, β-sheets are yellow, while undefined loops are gray. (**B**) Surface representation of SIRT5 (PDB code: 3RIY), highlighting the succinyl-lysine peptide substrate (Thr-Ala-Arg-SLL-Ser-Thr-Gly-Gly, red) and the NAD^+^ cofactor (green), bound in their respective pockets. (**C**) Magnified view of the two binding pockets, highlighting the ligand (red), NAD^+^ (green), and the surrounding amino acids (light yellow).

However, despite the structural similarity among all isoforms, only SIRT5 catalyzes the cleavage of negatively charged acylated substrates. SIRT5 is mainly located in the mitochondria (together with SIRT3 and SIRT4) and is highly expressed in the brain, heart, testis, and lymphoblasts. Its unique structure consists of fourteen α helices and nine β strands, organized in two domains: the Zn^2+^-binding domain and the Rossmann fold domain (Figure 1) [4]. Moreover, SIRT5 presents two binding pockets, one for the protein substrate and the other for the NAD^+^ cofactor, both located in the interspace between the Zn^2+^-binding domain and the Rossmann fold domain. Differently from other sirtuins, SIRT5 has a peculiar biochemistry and distinctive amino acid residues in the active site, namely Ala86, Tyr102, and Arg105. In the catalytic domain, the two non-hydrophobic residues, Tyr102 and Arg105, form hydrogen bonds and ionic-bond interactions with the carboxyl group of the succinyl-lysine substrate [5]. Furthermore, the presence of Ala86 makes the SIRT5 acyl-lysine binding pocket larger than that of other SIRTs.

SIRT5 is highly expressed in the mitochondrial matrix and has a specific mitochondrial localization sequence (MLS); however, it is also present in the cytosol. Recent studies have reported that an alternative splicing of the SIRT5 mRNA leads to two dominant isoforms, SIRT5^iso1^ and SIRT5^iso2^, containing the same N-terminal MLS, but differing at the C-terminal portion. While SIRT5^iso1^ was found only in the cytosol, SIRT5^iso2^ was also detected in the cytoplasm [5]. Moreover, two additional SIRT5 isoforms (SIRT5^iso3^ and SIRT5^iso4^) have been reported in the NCBI database; however, no information is available regarding their expression, localization, or functional properties [6].

**Figure 2:**
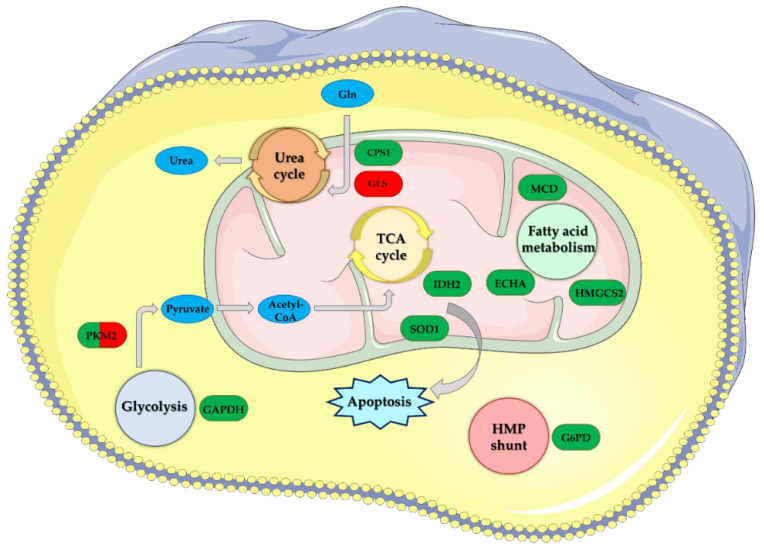
Graphical representation of the main metabolic pathways regulated by SIRT5. The activated targets are contained in green boxes, whereas inhibited proteins are in red boxes.

SIRT5 catalyzes the deacetylation, demalonylation, desuccinylation, and deglutarylation of lysine, modulating many metabolic enzymes by post-translational modifications (Figure 2). More specifically, it is involved in several cellular metabolic pathways, anti-inflammatory processes, antitumor mechanisms, and in the regulation of the response to oxidative stress [7].

The mitochondrial isoform of SIRT5 regulates ammonia metabolism, fatty acid oxidation, glycolysis, the tricarboxylic acid (TCA) cycle, the electron transport chain (ETC), and apoptosis [8]. Its specific substrates include carbamoyl phosphate synthetase 1 (CPS1) and glutaminase (GLS), which regulate the balance between the urea cycle and mitophagy. Furthermore, SIRT5 demalonylates glyceraldehyde 3-phosphate dehydrogenase (GAPDH) and other enzymes in the glycolytic cascade, leading to increased GAPDH activity. It desuccinylates pyruvate kinase M2 (PKM2) at Lys311 to increase pyruvate kinase activity, thereby promoting the glycolytic flux [3]. In contrast, Xiangyun and co-workers reported that SIRT5 desuccinylates PKM2 at Lys498 to inhibit its activity in tumor cells [9]. Additionally, SIRT5 is specifically involved in the pentose phosphate pathway, which allows the production of ribose-5-phosphate, a precursor for the synthesis of nucleotides. This cycle also promotes the reduction of NADP^+^ to NADPH, blocking ROS storage in cells and playing a role in the regeneration of the reduced form of GSH (an ROS scavenger). In detail, SIRT5 mediates the deglutarylation and consequent activation of glucose-6-phosphate-1-dehydrogenase (G6PD) in stress conditions to maintain cellular redox homeostasis [8]. Furthermore, SIRT5 regulates 3-hydroxy-3-methylglutaryl-CoA synthase 2 (HMGCS2), enoyl-CoA hydratase α-subunit (ECHA), and malonyl-CoA decarboxylase (MCD). The latter catalyzes the conversion of malonyl-CoA to acetyl-CoA and is implicated in the homeostasis of these metabolites in mitochondria and peroxisomes [10]. SIRT5 also desuccinylates superoxide dismutase [Cu-Zn] (SOD1) and isocitrate dehydrogenase 2 (IDH2) [11].

The cytosolic SIRT5 isoform acts as a glycolytic enzyme and interacts with the 40S and 60S ribosomal subunits during gene translation [5].

Most notably, SIRT5 is also involved in several intracellular signaling pathways, exerting a specific influence in the regulation of protein functions through their post-translational modification (PTM) in physiological and pathological conditions [12,13,14,15,16]. However, the functional role of SIRT5 in regulating many of its proposed targets has yet to be investigated.

## 2. SIRT5 Modulators

Due to the potential role of SIRT5 as a pharmacological target in cancer, diabetes, cardiovascular diseases, obesity, neurodegenerative disorders, and inflammation, many studies have been undertaken to identify new molecules acting as SIRT5 activators or inhibitors.

**Figure 3:**
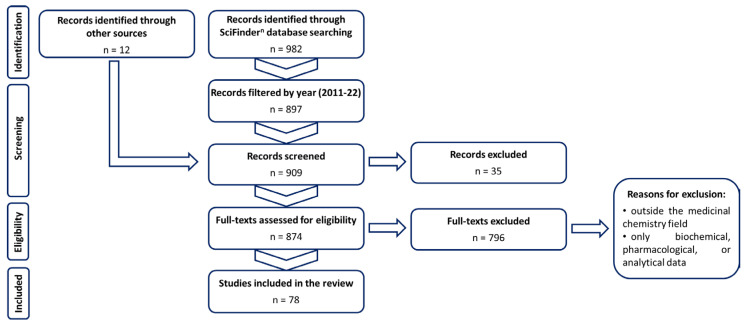
PRISMA flow diagram, describing the screening process that led to the selection of the works discussed in this review.

Here, we report a comprehensive overview of the SIRT5 modulators that have been described in the literature in the last decade. All selected molecules are classified as activators or inhibitors based on their effects on SIRT5, and further divided into natural derivatives and synthetic compounds. The screening process and the subsequent selection of the relevant literature sources for this review are described in Figure 3 through a PRISMA flow diagram.

The first part of the following two sections is dedicated to natural products (NPs), which represent an invaluable source of structurally unique bioactive compounds, and a paramount source of new drug candidates [17,18,19,20,21]. Semisynthetic and synthetic derivatives are described in the second part of both sections. The origin of the compounds, their biological activities and mode of action, as well as viable opportunities for their future development will be the focus of this work.

### 2.1. Activators

SIRT5 can be activated by sirtuin-activating compounds (STACs), which induce cellular and physiological effects amplified by downstream signaling pathways. These molecules could be useful in hyperlipidemia and hypercholesterolemia, in the prevention and treatment of type 2 diabetes [22], in the therapeutic approach for Alzheimer’s disease [23], or as medication for rheumatoid arthritis [24] and various tumors [14,25].

#### 2.1.1. Natural Compounds

**Figure 4:**
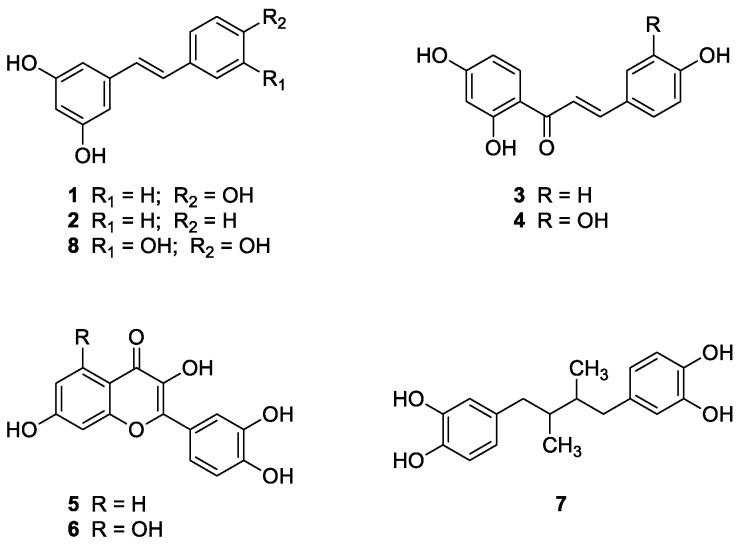
Chemical structures of the natural activators (**1**–**7**) of SIRT5.

In a 2006 patent application, Howitz et al. assayed a group of compounds, previously reported as SIRT1 activators, for their ability to activate SIRT5 [22]. Among the detected STACs, several were natural compounds; the most interesting are discussed in the following paragraph. Resveratrol (**1**) and pinosylvin (**2**) are natural stilbenes obtained from plants: **1** is commonly found in peanuts, grapes, raspberries, and vegetables and it is commonly used as a dietary supplement, whereas **2** is synthesized in plants during fungal infections, in response to stress and physical damage [26]. Isoliquiritigenin (**3**) and butein (**4**) are bioactive chalcone derivatives. Compound **3**, found in *Glycyrrhiza glabra*, is known to be capable of reducing oxidative damage in diabetic patients affected by neuropathy [27], whereas **4**, a compound found in several plants, including *Toxicodendron vernicifluum, Dahlia* spp., and *Butea* spp., was identified as an antioxidant agent involved in the control of oxidative stress and apoptosis. It was also described as a neuroprotective compound, supporting the correct cerebral activity via an increase in neuronal cell survival and the prevention of H_2_O_2_-mediated neurotoxicity [28]. Two flavone derivatives were also identified as STACs, namely fisetin (**5**), a natural flavonoid found in fruits, vegetables, and nuts, known for its antioxidant and anti-inflammatory properties [29], and quercetin (**6**), a polar auxin transport inhibitor (ATI), widely distributed in nature [30]. Finally, nordihydroguaiaretic acid (**7**), a natural antioxidant and anti-inflammatory lignan found in *Larrea tridentata*, showed activity on SIRT5 [31,32]. Compounds **1**–**7** were evaluated for their ability to activate SIRT5 at 200 µM; the results were expressed as the rate of activation with respect to the control (**1**: 4.95 ± 0.48-fold; **2**: 2.71 ± 0.092-fold; **3**: 3.93 ± 0.30-fold; **4**: 2.19 ± 0.10-fold; **5**: 2.61 ± 0.19-fold; **6**: 2.18 ± 0.10-fold; **7**: 1.91 ± 0.02-fold) [22]. The chemical structures of compounds **1**–**7** are reported in Figure 4. To summarize, the reported compounds can be classified into four major groups of polyphenols, namely stilbenes (**1**, **2**), chalcones (**3**, **4**), flavones (**5**, **6**), and lignans (**7**).

Compound **1** was also studied by Gertz et al., who evaluated its effects on SIRT5 and SIRT3 [33]. The biological analyses revealed a 2.5-fold stimulation of the deacetylase activity of SIRT5 against the fluorophore-modified peptide substrate ‘‘Fluor-de-Lys’’ (FdL) in response to 0.2 µM resveratrol (**1**). Compound **1** was also shown to inhibit the human SIRT3 and stimulate SIRT1. Unfortunately, **1** is characterized by low solubility, which does not allow full inhibition of SIRT3 and maximum stimulation of SIRT5 to be achieved. The low bioavailability of resveratrol (**1**) prompted the hypothesis that its metabolites might be responsible for the in vivo effects. The biological analysis of piceatannol (**8**, Figure 4), a metabolite of **1** showing higher solubility, revealed that the compound stimulates SIRT5 and inhibits SIRT3 (EC_50_ of 0.07 ± 0.02 mM for SIRT5) [33,34].

#### 2.1.2. Synthetic Compounds

**Figure 5:**
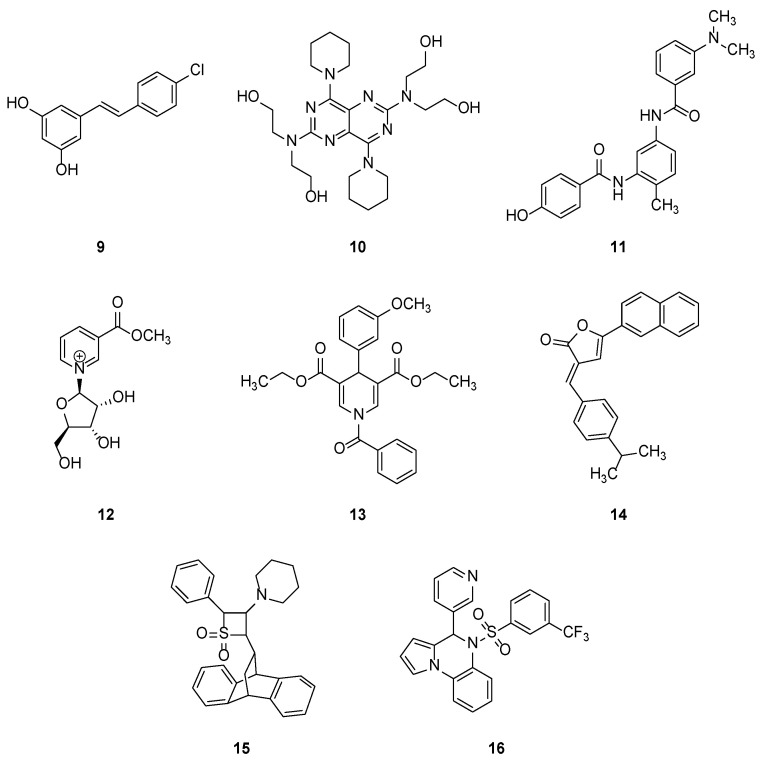
Chemical structures of the synthetic activators (**9**–**16**) of SIRT5.

In addition to the natural molecules reported in the previous section, Howitz et al. assayed several synthetic compounds, previously known as SIRT1 activators, on SIRT5 [22]. Most of the tested molecules were stilbene derivatives, including BML-217 (**9**, Figure 5), which showed the maximum stimulation of SIRT5 (13.6-fold). Interesting results were also obtained for dipyridamol (**10**, Figure 5; 8.56 ± 0.30-fold), a nucleoside transport and PDE3 inhibitor used in the prevention of blood clot formation [35], and for ZM336372 (**11**, Figure 5; 0.74 ± 0.05-fold), a c-Raf inhibitor [22,36].

Sauve et al. synthesized several nicotinamide riboside derivatives and assayed them on SIRT5 [37]. The most interesting result was found for **12** (Figure 5), which showed 80% activation at 600 µM [37].

More recently, Hu and co-workers studied the role of SIRT5 in pancreatic ductal adenocarcinoma (PDAC) and developed a small-molecule SIRT5 activator, MC3138 (**13**, Figure 5) [38]. The compound was tested on human PDAC cell lines, organoids, and PDX tumors. The results of the biochemical enzymatic assays indicated that **13** exhibited the selective activation of SIRT5 over SIRT1/3. The cell viability experiments showed that **13** reduced PDAC cell viability, with IC_50_ values ranging from 25.4 mmol/L to 236.9 mmol/L. The toxicity assays demonstrated that **13** combined with gemcitabine could be a safe and effective therapeutic option for PDAC characterized by low SIRT5 expression [38].

In a computational study, Schlicker et al. performed docking screening on a library of small molecules, using the crystal structures of human SIRT2/3/5/6 as models [39]. The virtual simulations suggested that CSC9 (**14**), CSC33 (**15**), and CSC38 (**16**) (Figure 5) could act as activators of SIRT5 [39].

### 2.2. Inhibitors

SIRT5 inhibitors (sirtuin-inhibiting compounds, STICs) have been studied for their potential role in the treatment of metabolic disorders [6,40], neurodegenerative pathologies [23,41,42], cardiovascular diseases [42,43], and cancer [3,44].

#### 2.2.1. Natural Compounds

**Figure 6:**
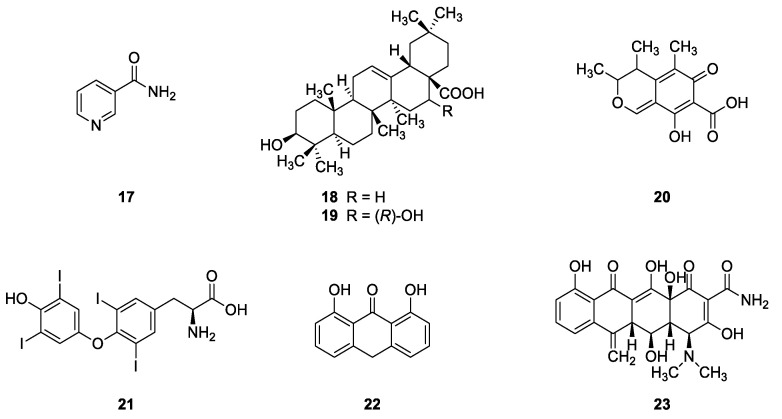
Chemical structures of the natural inhibitors (**17**–**23**) of SIRT5.

Nicotinamide (NAM, **17**, Figure 6) represents one of the major precursors for NAD biosynthesis. As a product of the reactions catalyzed by SIRTs, it acts as an endogenous non-competitive inhibitor of these enzymes, with an IC_50_ = 150 μM against SIRT5 [45].

In 2015, Huang et al. performed a screening on an NP database, discovering interesting SIRT5-inhibitory activity for oleanolic acid (**18**) and echinocystic acid (**19**) (Figure 6), with IC_50_ values of 70 and 40 μM, respectively [46]. Compound **18** is widely distributed in food (olive oil, garlic, etc.) and plants (*Phytolacca americana*, *Syzygium* spp., *Rosa woodsi,* etc.), and it exhibits hepatoprotective, antitumor, and antiviral properties. Compound **19** (Figure 6) is a triterpenoid derivative found in several plants, such as *Codonopsis lanceolata*, *Cucurbita foetidissima*, and *Eclipta alba*, and is known to have anti-inflammatory, antiviral, and anticancer activities [46].

Guetschow et al. identified several natural SIRT5 inhibitors via a high-throughput screening of a Prestwick Chemical library, comprising 1280 approved drugs [47]. Eight of them were selected as promising inhibitors of SIRT5, among which four were natural compounds: antymicin (**20**, a commercial piscicide used in catfish production), thyroxine (**21**, a thyroid medicine administered in the treatment of goiter and thyroid nodules), anthralin (**22**, a topical drug for the treatment of local diseases, such as psoriasis), and methacycline (**23**, a derivative of tetracycline used against Gram-positive, Gram-negative, and L-form bacteria) [47,48]. The IC_50_ values against SIRT5 were calculated as follows: **20**, IC_50_ = 90 μM; **21**, IC_50_ = 2.2 μM; **22**, IC_50_ = 0.1 μM; **23**, IC_50_ = 3.6 μM [48]. The structures of compounds **20**–**23** are reported in Figure 6.

#### 2.2.2. Synthetic Compounds

Over the years, several research groups have based the design of new SIRT5 inhibitors on the discovery that this isoform preferentially catalyzes the hydrolysis of succinyl and malonyl groups, rather than acetyl groups, from lysine residues. Most notably, SIRT5 is the only SIRT that exhibits this unique preference. Therefore, this characteristic has been conveniently exploited to develop selective inhibitors of SIRT5 [1].

**Figure 7:**
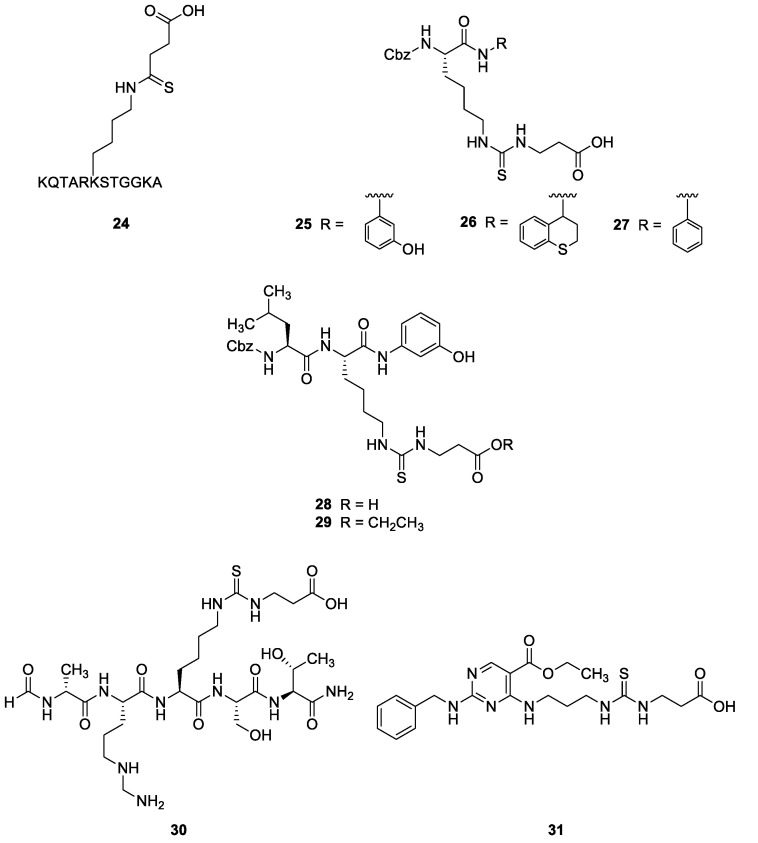
Chemical structures of the linear peptides (**24**–**31**) with inhibitory activity against SIRT5.

In 2012, He and co-workers reported for the first time the synthesis of a histone H3 lysine 9 (H3K9) thiosuccinyl peptide (H3K9Tsu, **24**, Figure 7) as a selective SIRT5 inhibitor [49]. They designed the molecule as a mechanism-based inhibitor, potentially capable of blocking the deacetylase activity of the enzyme by the formation of a stalled covalent intermediate. Compound **24** exhibited an IC_50_ of 5 μM against SIRT5 and was inactive (IC_50_ >100 μM) on other isoforms; the selectivity was achieved based on the inability of other SIRTs to recognize malonyl and succinyl lysine peptides. However, the compound was not active in whole-cell assays due to its low permeability [49]. Encouraged by these results, Lin reported the discovery of the first series of thiourea derivatives as potent inhibitors of SIRT5 [50]. The most interesting compounds, JH-I5-2 alias **25**, **26**, and **27**, exhibited IC_50_ values of 0.89, 0.45, and 12 μM, respectively [50]. These peptides became the starting point for a new class of analogues containing the thiourea moiety. In this context, Negròn Abril et al. developed cell-permeable SIRT5-selective inhibitors [51]. Among the synthesized derivatives, DK1-04 (**28**, Figure 7) showed the strongest inhibition, with an IC_50_ of 0.34 µM (no SIRT1-3,6 inhibition at 83.3 µM). Because **28** contained a free carboxylic acid, which could hinder cellular permeability, the function was protected with either an acetomethoxyl or an ethyl ester, which could be easily hydrolyzed in cells to release the active form of the compound. The prodrugs were also tested in 2D proliferation assays on MCF7 and MDA-MB-231 breast cancer cells: DK1-04e (**29**) showed the strongest inhibition of cell growth. The researchers also performed in vivo studies using a mouse model of breast cancer, MMTV-PyMT. The treatment with **29** (50 mg/kg, daily for 3 weeks) significantly reduced the tumor size and weight. Importantly, **29** did not cause any apparent toxicity or significant weight loss in mice. These results suggest that SIRT5 inhibitors have promising potential as innovative treatment options for breast cancer [51].

Many other research groups investigated thiourea derivatives, albeit with more modest success. In 2016, Liu and co-workers synthesized peptide **30** (Figure 7) [52], characterized by a central N^ε^-carboxyethyl-thiocarbamoyl-lysine residue. Compound **30** showed an IC_50_ of 7.6 ± 1.5 μM against SIRT5 and very weak inhibitory activity against SIRT1/3/6 (IC_50_ > 1000 μM). Unfortunately, its inhibition of SIRT2 was found to be only around 13-fold weaker than that of SIRT5 (IC_50_ = 96.4 ± 18.5 μM). The same research group also synthesized some cyclic derivatives of **30**, which are reported in the next section [52]. Recently, Yang et al. prepared a series of 3-thioureidopropanoic acid derivatives mimicking glutaryl-lysine substrates [53]; among them, **31** (Figure 7) showed promising inhibitory activity and selectivity for SIRT5 (IC_50_ = 3.0 μM, SIRT1-3,6 IC_50_ > 600 μM) [53].

**Figure 8:**
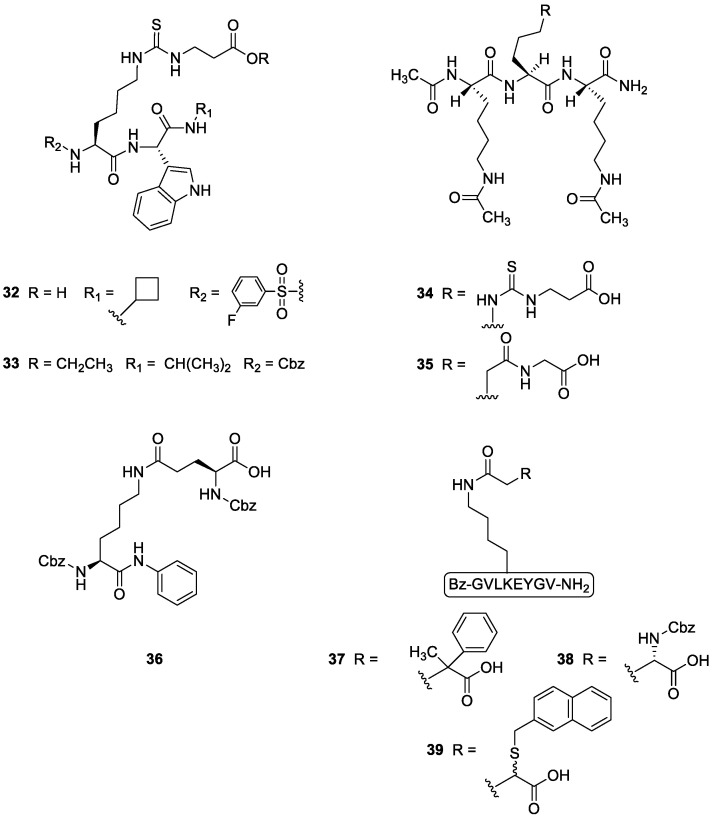
Chemical structures of the linear peptides (**32**–**39**) with inhibitory activity against SIRT5.

Among a series of compounds bearing the thiourea warhead reported by Rajabi et al., **32** (Figure 8) exhibited the most potent and selective SIRT5-inhibitory activity (IC_50_ = 0.11 μM) [54]. This peptide became the lead compound for a series of optimization campaigns, carried out by different research groups. In 2022, Rajabi and co-workers investigated isostere and prodrug derivatives of **32**, modified at the carboxylic function [55]. In a BioRxiv preprint, Bolding et al. reported the study of fluorosulfate analogues, with potential activity in vivo [56]. From the same class, Yan and co-workers identified a prodrug, NRD167 (**33**, Figure 8), which was able to block the proliferation of the SIRT5-dependent cell lines SKM-1 and OCI-AML2 (IC_50_ of 5 and 8 μmol/L, respectively), and to induce >80% apoptosis (at 5 and 10 μmol/L, respectively) [57]. Zang et al. identified the small peptide **34** (Figure 8), endowed with modest activity on SIRT5 (IC_50_ = 5 μM) and high selectivity vs. SIRT1 and 6 [58]. In the same year, He and co-workers discovered weaker activity for the analogue **35** (Figure 8, IC_50_ = 92.1 ± 3.5 μM), while studying the mechanism of action of several pan-SIRT1-3 derivatives bearing an l-2-amino-7-carboxamidoheptanoic acid (l-ACAH) moiety [59].

In 2015, Polletta and co-workers investigated the effect of SIRT5 on ammonia detoxification [60]. Their results showed that ammonia production increases in SIRT5-silenced and decreases in SIRT5-overexpressing cells. They also obtained the same ammonia increase when using a new specific inhibitor of SIRT5, MC3482 (**36**, Figure 7). Compound **36** inhibited the desuccinylase activity of SIRT5 (42% inhibition at 50 μM), affecting glutamine metabolism by interaction with the GLS catalytic domain. Although it was quite selective and did not inhibit SIRT1/3, it exhibited low in vitro potency [60].

A campaign to design analogues of CPS1, a substrate of SIRT5, was undertaken by Roessler and co-workers [61]. Among their peptide derivatives, the most promising was **37** (Figure 8), which showed a K*_i_* value of 4.3 μM against SIRT5 and good results in the selectivity assays (K*_i_* > 50 μM against SIRT1/2/3) [61]. Moreover, **38** (Figure 8) exhibited significant inhibition, with a K*_i_* of 38.1 ± 0.63 μM [61]. In a similar effort, Kalbas et al. developed several compounds related to CPS1, substituted at position 3 of the succinyl moiety; among them, **39** (Figure 8) showed very potent SIRT5 inhibition (IC_50_ = 15.4 nM) [62].

Peptide macrocyclization is an efficient approach to confer upon a linear peptide enhanced metabolic stability and cell permeability. Therefore, the synthesis of cyclic peptides from linear sequences could be an interesting technique for the development of innovative SIRT5 inhibitors.

**Figure 9:**
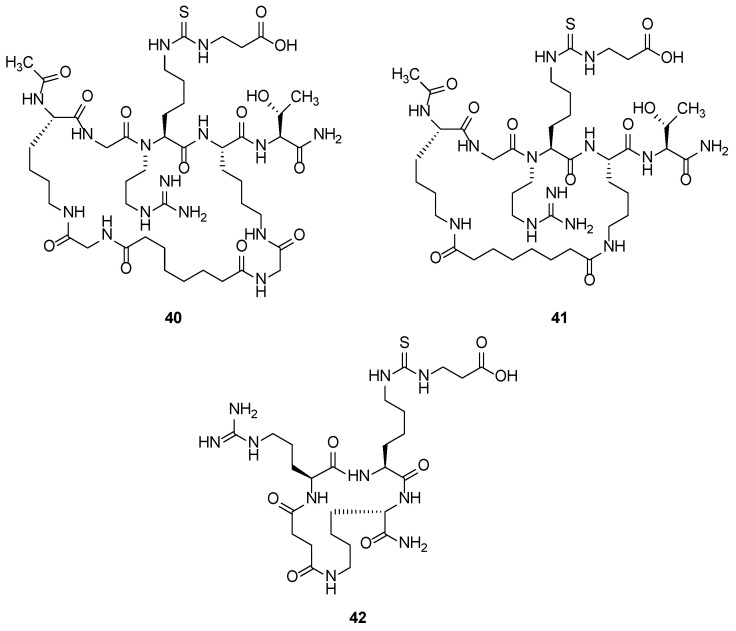
Chemical structures of the cyclic peptides (**40**–**42**) with inhibitory activity against SIRT5.

On these premises, Liu et al. synthesized the cyclic derivatives **40** and **41** (Figure 9) of the linear peptide **30** (Figure 7) [52]. These compounds exhibited comparable inhibitory potency to that of the parent peptide (**40**, IC_50_ = 6.0 ± 3.0 μM; **41**, IC_50_ = 7.5 ± 4.0 μM), suggesting that the macrocyclic bridging units in **40** and **41** were unable to constrain the peptide backbone into a bioactive conformation, or interfered with the binding of the compounds to the SIRT5 active site. Interestingly, the same macrocyclic bridging units were able to significantly enhance the potency of a similar linear peptide against SIRT1/2/3/6 [52,63,64].

Later, Jiang and co-workers prepared several cyclic tripeptides endowed with higher activity with respect to **40** and **41** [65]. Among them, **42** (Figure 9) exhibited the highest activity and selectivity vs. SIRT5 (IC_50(SIRT5)_ = 2.2 ± 0.89 μM; IC_50(SIRT1)_ = 254.2 ± 32.4 μM; IC_50(SIRT2)_ = 131.3 ± 46.5 μM; IC_50(SIRT3)_ > 450 μM; IC_50(SIRT6)_ > 1000 μM) [65].

Peptide derivatives are usually characterized by low biostability and scarce membrane permeability; therefore, the identification of small molecules is crucial to overcome these limits.

**Figure 10:**
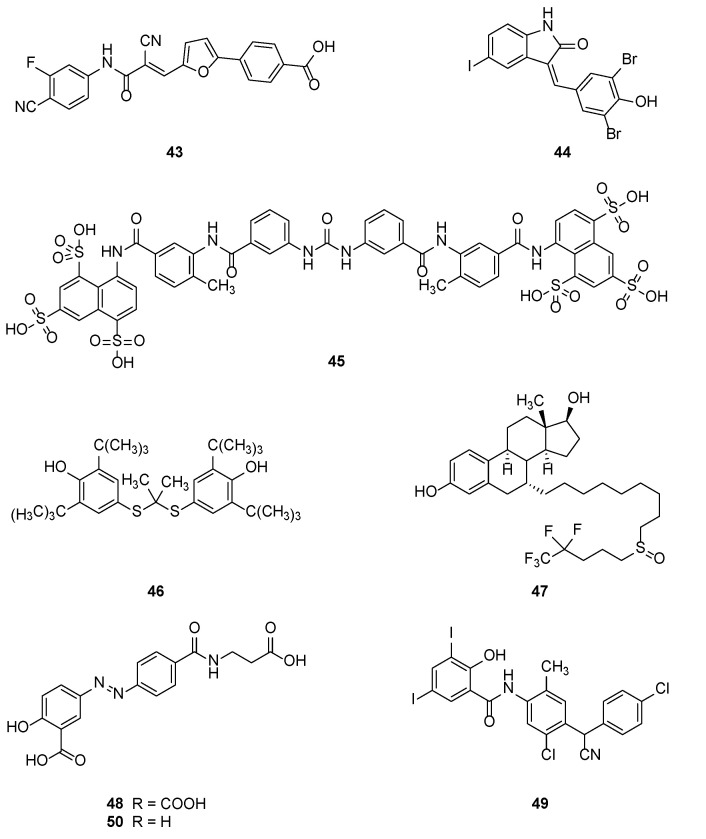
Chemical structures of the synthetic derivatives (**43**–**50**) with inhibitory activity against SIRT5.

Liu and co-workers synthesized a library of (*E*)-2-cyano-*N*-phenyl-3-(5-phenylfuran-2-yl)acrylamide derivatives, among which **43** (Figure 10) emerged as the most potent inhibitor of SIRT5, with an IC_50_ of 5.59 ± 0.75 μM [66]. Further biochemical studies revealed that **43** likely acts by competing with the succinyl-lysine substrate for the ligand-binding site of SIRT5. The compound also exhibited promising selectivity for SIRT5 over SIRT2/6 [66].

While verifying the cross-reactivity of a series of SIRT inhibitors towards SIRT5, Suenkel et al. found that GW5074 (**44**, Figure 10), a known SIRT2 blocker, showed interesting activity against SIRT5 [67]. The effect of **44** on SIRT5 deacetylation was comparable to that exerted on SIRT2 (more than 40% inhibition at 12.5 μM). However, **44** was also active against kinases; hence, it was deemed unsuitable for further in vivo studies. Nonetheless, it remains a promising starting point for the development of specific SIRT5 inhibitors [67].

Suramin (**45**, Figure 10), a drug approved for the treatment of human sleeping sickness caused by trypanosomes, was identified by Schuetz A. et al. as an inhibitor of SIRT5, with an IC_50_ value of 22 μM [68]. Two crystal structures of SIRT5, one in complex with ADP-ribose and the other with suramin (**45**), were reported by the authors. The analysis of the structures revealed that **45** acts as a linker molecule, resulting in the dimerization of SIRT5. This finding may lead to the development of a new class of inhibitors that not only bind specifically to the active site of the enzyme, but also function as linker molecules, thus limiting enzyme mobility and accessibility. Unfortunately, the fact that suramin (**45**) also targets the NAD^+^-binding region of SIRT5 makes it non-selective for similar binding pockets. Indeed, **45** has been reported to inhibit various other NAD^+^/NADP^+^-dependent enzymes, such as the lactate dehydrogenase from *Dirofilaria immitis* and *Onchocerca volvulus*, and the glyceraldehyde-3-phosphate dehydrogenase from *Trypanosoma brucei*. This highlights that the most efficient strategy to develop selective SIRT inhibitors is to target the substrate-binding site, and not the NAD^+^-binding pocket, in order to avoid secondary effects [68].

Among the compounds identified by Guetschow and co-workers via the high-throughput screening approach described in the previous section, four synthetic approved drugs exhibited promising inhibitory activity vs. SIRT5 [47]. Probucol (**46**), an anti-hyperlipidemic drug, showed an IC_50_ of 1.6 μM; fulvestrant (**47**), an estrogen receptor antagonist used in hormonal therapy, had an IC_50_ of 2.6 μM; balsalazide (**48**), a drug used for the treatment of inflammatory bowel disease, exhibited an IC_50_ of 3.9 μM; finally, closantel (**49**), a veterinary drug with potent antiparasitic activity, displayed an IC_50_ of 2.7 μM [47,48]. The chemical structures of compounds **46**–**49** are reported in Figure 10. Inspired by this study, Glas et al. selected balsalazide (**48**) as the starting point for the development of new synthetic derivatives [48]. Unfortunately, none of the analogues showed improved inhibitory activity with respect to the parent compound. In detail, changes to the *N*-aroyl-β-alanine side chain disrupted the activity, while the introduction of a truncated salicylic acid moiety minimally altered the potency. The most interesting derivative was **50** (Figure 10), which showed a minimal loss of activity, demonstrating that modifications on the salicylic acid portion are tolerated, to some extent (SIRT5 inhibition = 73% at 50 μM). Interestingly, the biological assays proved that **48** and its derivatives were selective for SIRT5 over SIRT1/2/3. Unfortunately, **48** cannot be considered as an optimal candidate for the inhibition of SIRT5, due to its minimal absorption from the gut, poor water solubility, and instability to enzymatic degradation by the intestinal microflora (reductive cleavage of the azo moiety) [48].

**Figure 11:**
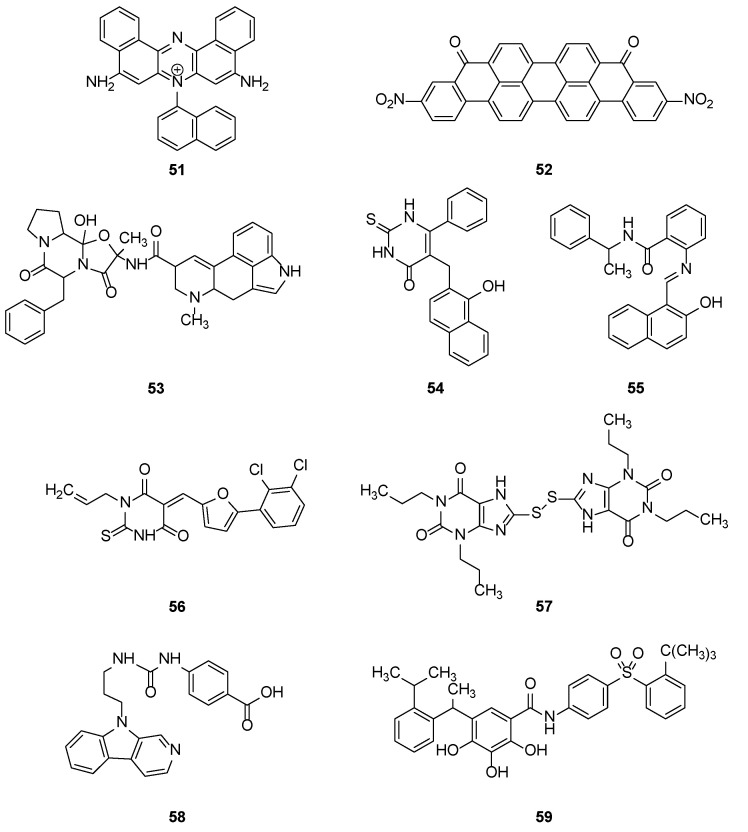
Chemical structures of the synthetic derivatives (**51**–**59**) with inhibitory activity against SIRT5.

As previously discussed, Schlicker and colleagues performed docking screening on a library of small molecules [39]. Several potential SIRT ligands were identified, but further activity tests were conducted only against SIRT2. Docking studies suggested that CSC1 (**51**), CSC14 (**52**), and CSC21 (**53**) could act as inhibitors of different isoforms of SIRT, including SIRT5 (Figure 11) [39].

The research group of Maurer and colleagues worked on the identification of small molecules as SIRT5 inhibitors; the compounds showed good potencies, but very poor selectivity vs. other SIRT isoforms [69]. In detail, two naphthol derivatives, cambinol (**54**) and sirtinol (**55**), already known for their anti-inflammatory properties [70,71], were proven to possess non-selective activity against SIRT5 at submicromolar concentrations (Figure 11) [69]. Moreover, they prepared several potent thiobarbiturate derivatives, which were also active against SIRT1/2. However, they were shown to be selective over SIRT3, an isoform that is co-localized with SIRT5. The thiobarbiturate derivative **56** (Figure 11) exhibited the highest inhibitory activity against SIRT5 (IC_50(SIRT1)_ = 5.3 ± 0.7 μM, IC_50(SIRT2)_ = 9.7 ± 1.6 μM, SIRT3: 41% inhibition at 50 μM, IC_50(SIRT5)_ = 2.3 ± 0.2 μM) [69]. Other potent SIRT5 inhibitors lacking the desired selectivity were synthesized by Han et al., based on the 8-mercapto-3,7-dihydro-1*H*-purine-2,6-dione scaffold [72]. The structure of one of the most potent derivatives (**57**) is reported in Figure 11 (IC_50(SIRT1)_ = 0.12 ± 0.01 μM, IC_50(SIRT2)_ = 1.19 ± 0.06 μM, IC_50(SIRT3)_ = 0.54 ± 0.05 μM, IC_50(SIRT5)_ = 0.39 ± 0.03, IC_50(SIRT6)_ = 128.7 ± 16.1 μM) [72].

Several new 9-substituted norharmane derivatives were studied by Yang and co-workers as SIRT5 inhibitors; the most active candidate, **58** (Figure 11), showed 35 and 52% inhibition at 30 μM and 100 μM, respectively [73]. This series was subsequently developed into a library of derivatives characterized by different linker bridges and phenyl substituents, which exhibited significant activities [74]. During the development of assay platforms based on fluorogenic substrates to identify SIRT inhibitors, the same group discovered that TW-37 (**59**, Figure 11), a Bcl-2 inhibitor, was capable of inhibiting SIRT5 in the low micromolar range (IC_50_ = 6 μM) [75].

## 3. Conclusions

Because SIRTs play essential roles in cell signaling pathways, gene translation, metabolism, and oxidative stress control, they are involved in several pathological conditions, including cancer, diabetes, and neurodegenerative and cardiovascular diseases. Specifically, SIRT5 is an attractive enzyme that not only catalyzes deacetylation reactions, but also exhibits strong demalonylase, desuccinylase, and deglutarylase activities. Consequently, SIRT5 is considered to be a promising molecular target for the treatment of several human diseases.

To date, the number of SIRT5 inhibitors reported in the literature far exceeds that of SIRT5 activators. Moreover, several known compounds and drugs have been identified as SIRT5 modulators by various screening approaches; however, some of them are non-selective and certain ones are considered pan-SIRT modulators (e.g., compounds **1**, **17**, **54**–**56**, etc.).

Overall, despite the promising results achieved in SIRT research and the potential of recent perspectives concerning the role of SIRT5 as a pro-viral factor [76] and as a catalyst in peptide self-assembly [77], it is necessary to consider the issue of selectivity. This is especially crucial because some SIRTs are co-present in the same cellular compartment (for example, SIRT5 and SIRT3 are both localized in the mitochondrial matrix). Furthermore, SIRT5 has pleiotropic roles in tumorigenesis, acting as a tumor suppressor or an oncogene via post-translational modifications, depending on cell conditions [6,44,78]. Hence, the balance between its activation and inhibition should be carefully considered in the development of a SIRT5 modulator.

In conclusion, SIRT5 is an interesting molecular target, and the regulation of its activity has many potential therapeutic applications for a variety of medical conditions. However, additional studies are crucial to identify the molecular determinants to achieve a selective effect, and to verify the feasibility of future pharmaceutical applications.
